# *Nocardia* brain abscess in a patient with chronic lymphocytic leukemia

**DOI:** 10.1590/0037-8682-0634-2021

**Published:** 2022-02-25

**Authors:** Guilherme Soares de Oliveira Wertheimer, Guilherme Duffles, Fabiano Reis

**Affiliations:** 1 Universidade Estadual de Campinas, Faculdade de Ciências Médicas, Departamento de Radiologia, Campinas, SP, Brasil.; 2 Universidade Estadual de Campinas, Departamento de Clínica Médica, Serviço de hematologia, Hemocentro, Campinas, SP, Brasil.

A 63-year-old man from southeastern Brazil was diagnosed in 2017 with chronic lymphocytic leukemia (CLL), Binet stage C. Four cycles of chemotherapy (fludarabine, cyclophosphamide, and rituximab) resulted in complete remission; however, recurrence occurred 42 months later. In April 2021, the patient was admitted because of a reduced level of consciousness. He presented with aphasia, right hemiparesis, and disorientation in the previous week. Magnetic resonance imaging (MRI) revealed an expansible lesion in the parietal lobe (Figure 1). The pattern was consistent with a pyogenic abscess that was drained, and investigation of the abscess material subsequently yielded positive cultures for *Nocardia cyriacigeorgica*. Treatment involved trimethoprim-sulfamethoxazole, ceftriaxone, and oxacillin, with good outcomes, without neurological deficits. 

**FIGURE 1: Axial:**
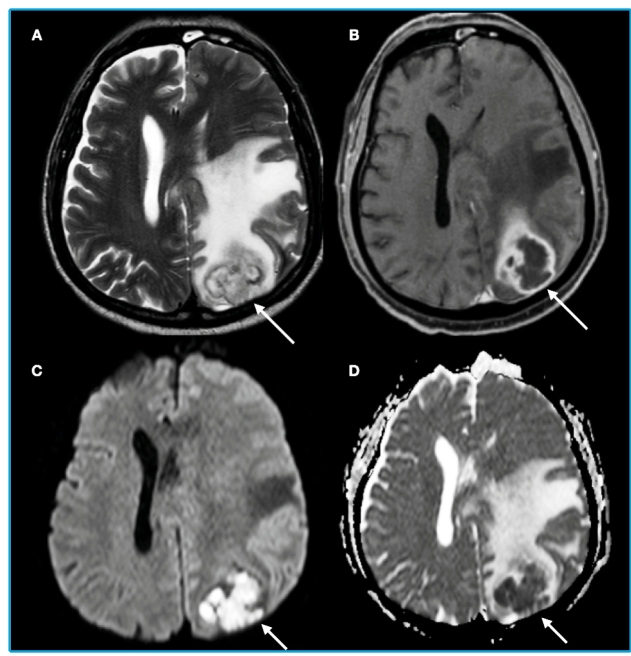
T2 weighted image (A), T1 weighted image after contrast administration (B), diffusion-weighted imaging (DWI) (C) and apparent diffusion coefficient (ADC) map (D), showing expansible lesion in the left parietal lobe, with peripheral ring enhancement (B, arrow), restricted diffusion, characterized by hyperintensity on DWI (arrow, C) and hypointensity on ADC map (D, arrow) and perilesional edema (A).

CLL involves alterations in cellular and humoral immunity. Nocardiosis is an opportunistic disease that frequently affects immunocompromised patients, especially those with problems related to cellular immunity, for example, a T cell deficiency associated with leukemia or alterations related to chemotherapy with compounds such as fludarabine^1^ ^,^ ^2^. The lung is the primary organ affected, but the central nervous system (CNS) is also infected in a third of cases and is frequently associated with an acute, rapidly progressive presentation^3^. The most frequent finding is brain abscess^3^, hence, MRI is useful for diagnosis. The MRI pattern is pathognomonic of a pyogenic abscess and involves single or multiple lesions with ring contrast enhancement and central necrosis with restricted diffusion. Nocardiosis should be considered in leukemia patients with brain abscess, and MRI allows for earlier detection, leading to earlier therapeutic intervention with a better clinical outcome.

## References

[1] Anaissie EJ, Kontoyiannis DP, O'Brien S, Kantarjian H, Robertson L, Lerner S (1998). Infections in patients with chronic lymphocytic leukemia treated with fludarabine. Ann Intern Med.

[2] Morrison VA (2007). Management of infectious complications in patients with chronic lymphocytic leukemia. Hematology Am Soc Hematol Educ Program.

[3] Barata CH, Oliveira DAG, Colombo AL, Pereira CAP (2000). Brain abscess by Nocardia sp in immunocompromised patient. Rev Soc Bras Med Trop.

